# Synthesis, crystal structure and Hirshfeld surface analysis of 3-(4-fluoro­phen­yl)-2-formyl-7-methyl­imidazo[1,2-*a*]pyridin-1-ium chloride monohydrate

**DOI:** 10.1107/S2056989023007272

**Published:** 2023-09-12

**Authors:** Firudin I. Guseinov, Viacheslav O. Ovsyannikov, Pavel V. Sokolovskiy, Yurii L. Sebyakin, Aida I. Samigullina, Mehmet Akkurt, Sevim Türktekin Çelikesir, Ajaya Bhattarai

**Affiliations:** a Kosygin State University of Russia, 117997 Moscow, Russian Federation; b N. D. Zelinsky Institute of Organic Chemistry, Russian Academy of Sciences, 119991 Moscow, Russian Federation; c MIREA, Russian Technology University, Lomonosov Institute of Fine Chemical Technology, Moscow 119571, Russian Federation; d Department of Physics, Faculty of Sciences, Erciyes University, 38039 Kayseri, Türkiye; e Department of Physics, Faculty of Science, Erciyes University, 38039 Kayseri, Türkiye; f Department of Chemistry, M.M.A.M.C. (Tribhuvan University), Biratnagar, Nepal; Texas A & M University, USA

**Keywords:** crystal structure, imidazo[1,2-*a*]pyridin-1-ium, hydrogen bonds, π–π inter­actions, Hirshfeld surface analysis

## Abstract

In the title salt, C_15_H_12_FN_2_O^+^·Cl^−^·H_2_O, the imidazo[1,2-*a*]pyridin-1-ium ring system of the cation is almostly planar [maximum deviaition = −0.047 (2) Å for the ring C atom with the attached arene ring] and forms a dihedral angle of 61.81 (6)° with the plane of the fluoro­phenyl ring. In the crystal, water mol­ecules form an *R*_2_^4^(8) motif parallel to the (100) plane by bonding with the chloride ions *via* O—H⋯Cl hydrogen bonds. The cations are connected along the *b* axis *via* N—H⋯O hydrogen bonds involving the O atoms of water mol­ecules, and C—H⋯O, C—H⋯Cl and π–π inter­actions [centroid-to-centroid distance = 3.6195 (8) Å] form layers parallel to the (100) plane. Furthermore, these layers are connected *via* π–π inter­actions [centroid-to-centroid distance = 3.8051 (9) Å] that further consolidate the crystal structure.

## Abstract 2

In the salt 3-(4-fluoro­phen­yl)-2-formyl-7-methyl­imidazo[1,2-*a*]pyridin-1-ium chloride monohydrate, water mol­ecules form an 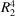 (8) motif parallel to the (100) plane by bonding with the chloride ions *via* O—H⋯Cl hydrogen bonds. The cations are connected along the *b* axis *via* N—H⋯O hydrogen bonds involving the O atoms of water mol­ecules.

## 1. Chemical context

**Figure 1:**
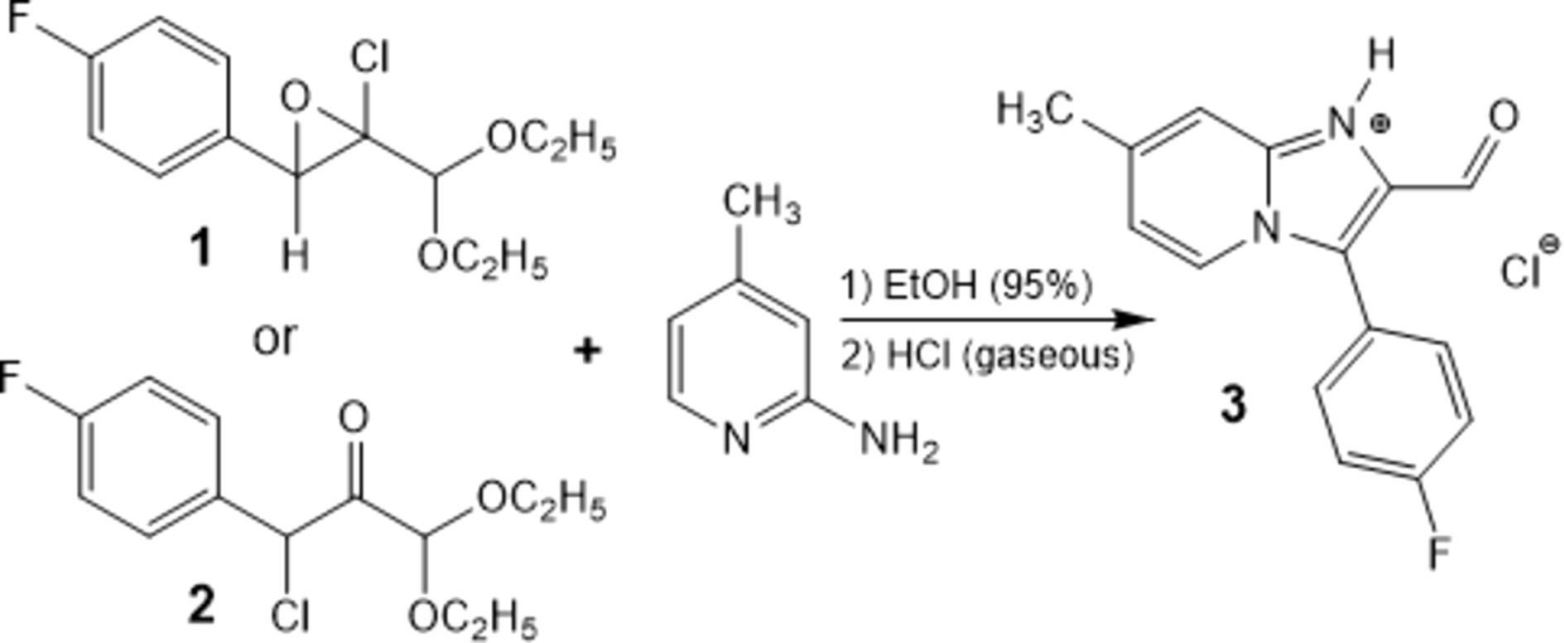
Reaction mechanism of the title compound.

Imidazo[1,2-*a*]pyridine is considered to be the most important derivative in the imidazo­pyridine system, with many important biological activities (Ribeiro *et al.*, 1998 ▸; Khalilov *et al.*, 2021 ▸). These derivatives exhibit a number of inter­esting properties, such as anti­cancer, anti­fungal, anti-inflammatory, anti­bacterial, anti­protozoal, anti­pyretic and anti-infective, as well as analgesic and pain relief and sedative properties (Ribeiro *et al.*, 1998 ▸; Almirante *et al.*, 1965 ▸; Safavora *et al.*, 2019 ▸). Imidazo[1,2-*a*]pyridine is present in various pharmaceutical products, such as zolpidem (used to treat insomnia), alpidem (sedative) (Lacerda *et al.*, 2014 ▸), zolimidine (used to treat peptic ulcers) (Tyagi *et al.*, 2012 ▸; Martins *et al.*, 2017 ▸), olprinone (acute heart failure), saripidem (sedative), necopidem (sedative), soraprazan, miroprofen and minodronic acid (Kielesiński *et al.*, 2015 ▸). Due to its importance in the pharmaceutical industry, much effort has been devoted to this heterocycle in order to develop an efficient, feasible and low-cost synthesis of imidazo[1,2-*a*]pyridine derivatives (Ribeiro *et al.*, 1998 ▸). Besides their biological activity, the transition-metal complexes of imidazole ligands have been found to possess a wide variety of functional properties, for example, as catalysts, supra­molecular building blocks, analytical reagents, *etc*. (Gurbanov *et al.*, 2020*a* ▸,*b* ▸; Kopylovich *et al.*, 2011 ▸; Mahmudov *et al.*, 2010 ▸, 2012 ▸). By the functionalization of the imidazole synthon their functional properties can be improved (Gurbanov *et al.*, 2022 ▸; Mahmoudi *et al.*, 2017*a* ▸,*b* ▸, 2019 ▸). In addition, the functional groups on the imidazole ring can participate in various types of inter­molecular inter­actions (Mahmudov *et al.*, 2022 ▸). Acetal-containing 2-chloro-2-(di­eth­oxy­meth­yl)-3-(4-fluoro­phen­yl)oxirane (**1**) or 1-chloro-3,3-dieth­oxy-1-(4-fluoro­phen­yl)propan-2-one (**2**) in reactions with bi- and polyfunctional nucleophiles (Fig. 1 ▸) turned out to be convenient in the mol­ecular design of various heterocyclic systems, in particular, heterocyclic carbaldehydes and their derivatives (Guseinov *et al.*, 1994 ▸, 1995 ▸, 1998 ▸, 2006 ▸, 2017 ▸, 2020 ▸; Pistsov *et al.*, 2017 ▸). We have found that electrophilic reagents (**1** or **2**) react with 2-amino-4-methyl­pyridine under certain conditions to transform into 3-(4-fluoro­phen­yl)-2-formyl-7-methyl­imidazo[1,2-*a*]pyridin-1-ium chloride (**3**) whose structure has been determined by NMR spectroscopy and X-ray diffraction methods (Fig. 1 ▸). 
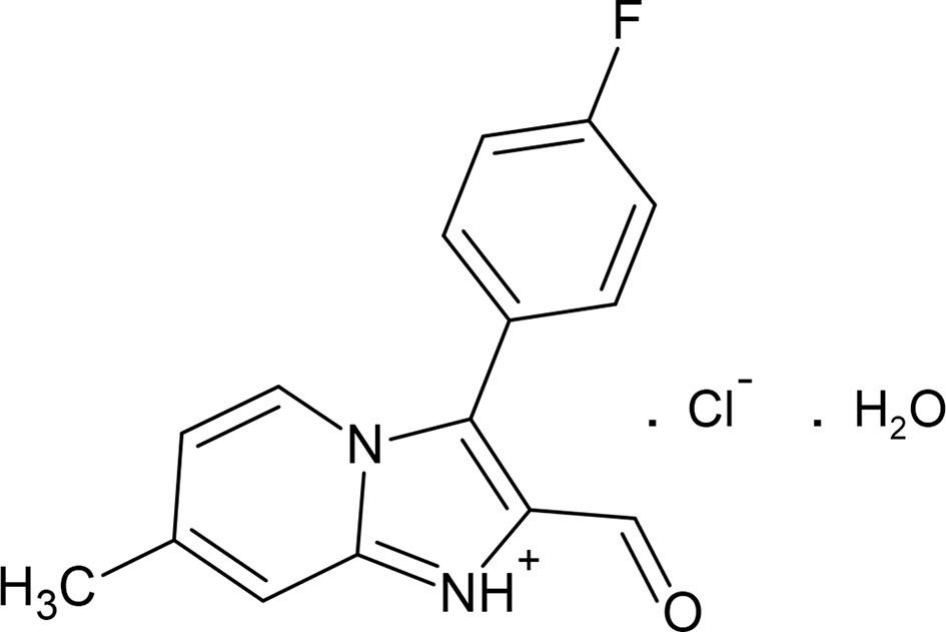


## 2. Structural commentary

**Figure 2:**
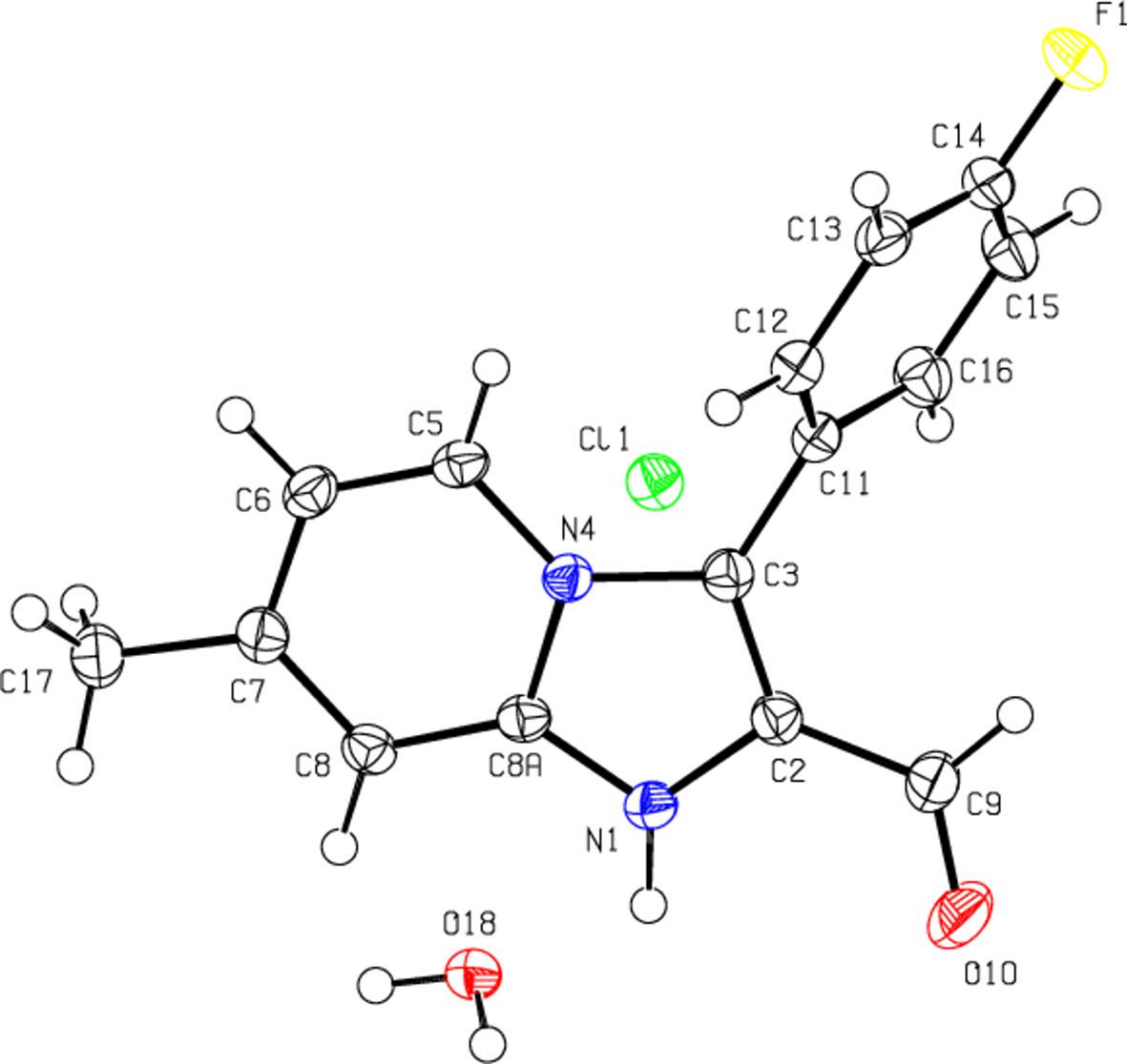
The mol­ecular structure of the title compound, showing the atom labelling and displacement ellipsoids drawn at the 50% probability level.

In the title salt (Fig. 2 ▸), the imidazo[1,2-*a*]pyridin-1-ium ring system (atoms N1/N4/C2/C3/C5–C8/C8*A*) of the cation is almost planar [maximum deviaition = −0.047 (2) Å for atom C3] and forms a dihedral angle of 61.81 (6)° with the plane of the fluoro­phenyl ring (C11–C16).

The bond lengths and angles in the mol­ecule of the title salt are comparable with those of closely related structures detailed in Section 4 (*Database survey*).

## 3. Supra­molecular features and Hirshfeld surface analysis

**Figure 3:**
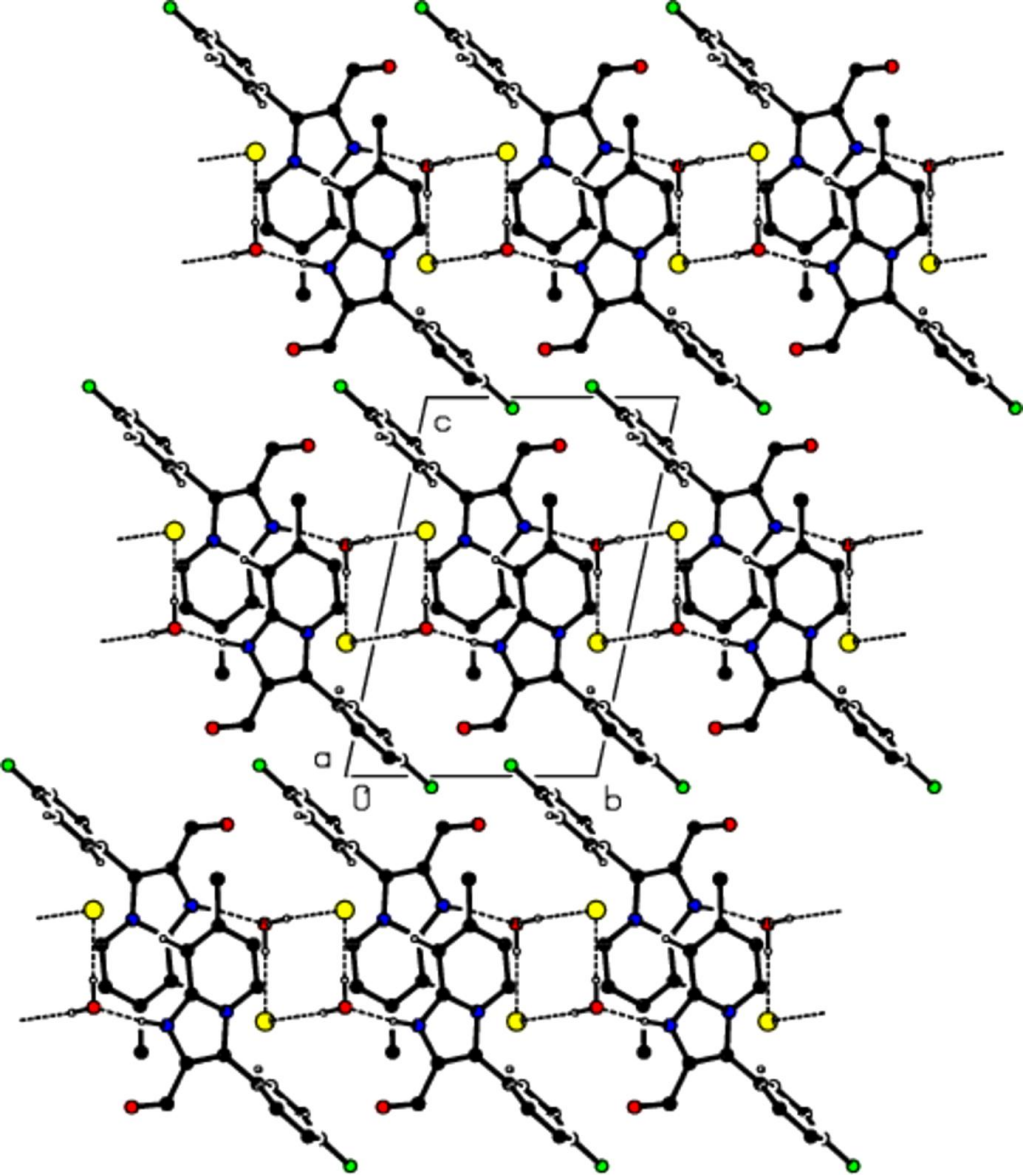
View of the mol­ecular packing along the *a* axis. N—H⋯O and O—H⋯Cl hydrogen bonds are shown as dashed lines.

**Figure 5:**
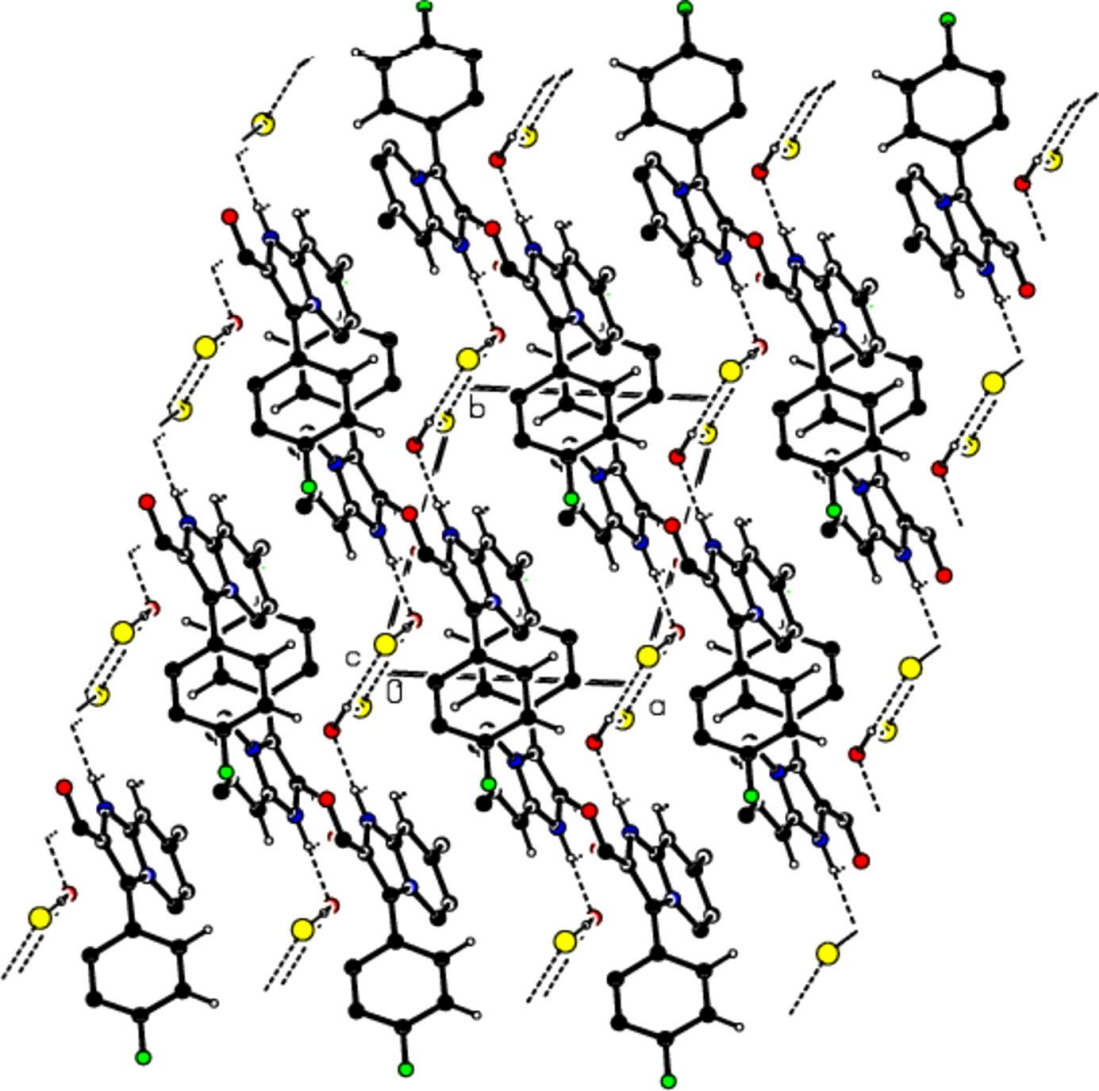
View of the mol­ecular packing along the *c* axis. Hydrogen bonds are depicted as in Fig. 3 ▸.

**Figure 6:**
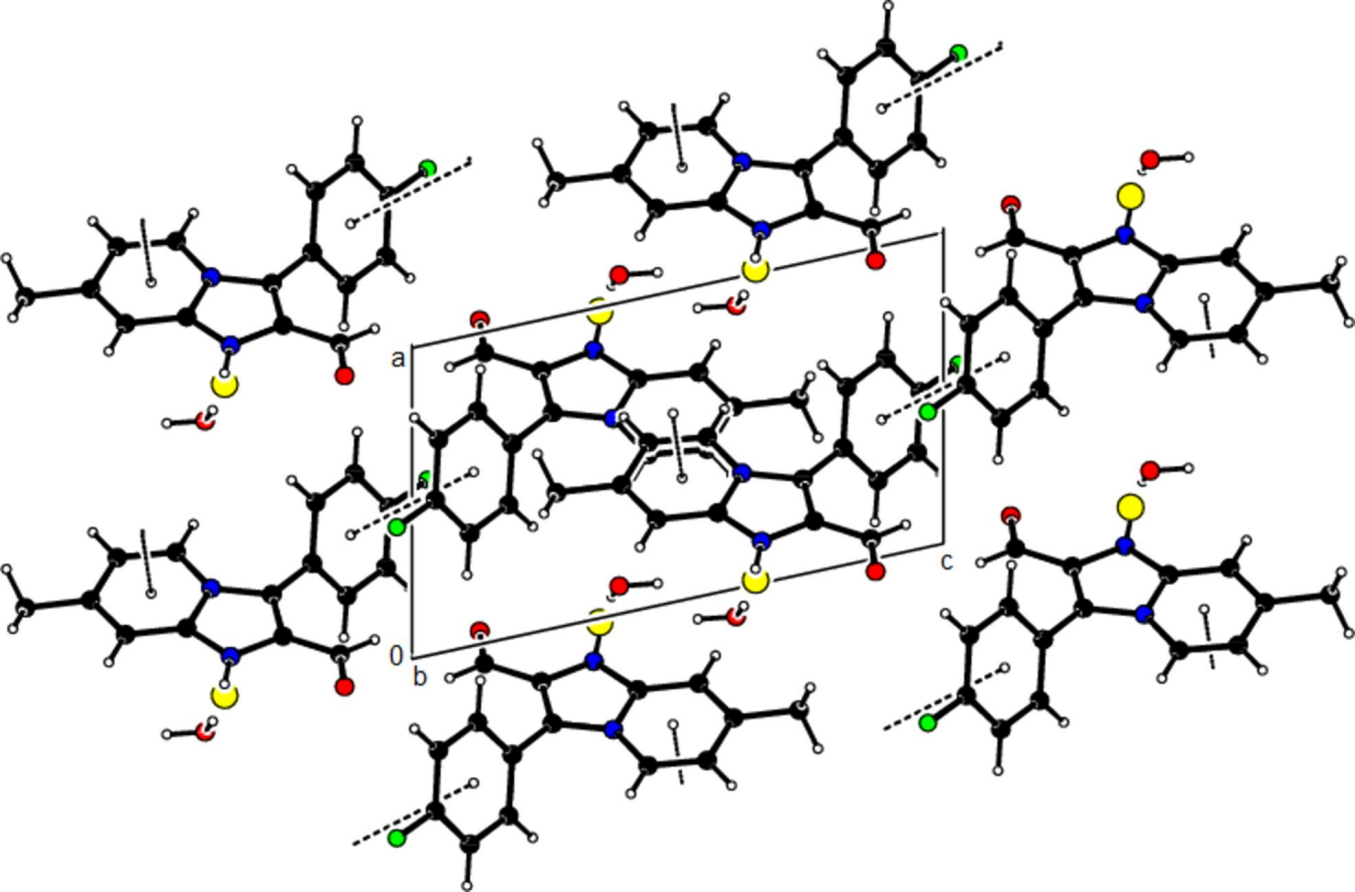
View of the π–π stacking inter­actions along the *b* axis in the unit cell.

In the crystal, water mol­ecules form an 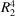 (8) motif (Bernstein *et al.*, 1995 ▸) parallel to the (100) plane by bonding with the chloride ions *via* O—H⋯Cl hydrogen bonds (Table 1 ▸ and Figs. 3 ▸ and 4 ▸). The cations are also connected along the *b* axis *via* N—H⋯O hydrogen bonds involving the O atoms of the water mol­ecules, and C—H⋯O, C—H⋯Cl and π–π inter­actions [*Cg*2⋯*Cg*2^iv^ = 3.6195 (8) Å; symmetry code: (iv) −*x* + 1, −*y* + 1, −*z* + 1; *Cg*2 is a centroid of the six-membered ring (N4/C5–C8/C8*A*) of the imidazo[1,2-*a*]pyridin-1-ium ring system (N1/N4/C2/C3/C5–C8/C8*A*)] form layers parallel to the (100) plane (Fig. 5 ▸). Furthermore, these layers are connected to each other *via* π–π inter­actions [*Cg*3⋯*Cg*3^vii^ = 3.8051 (9) Å; symmetry code: (vii) −*x* + 1, −*y*, −*z* + 2; *Cg*3 is a centroid of the fluoro­phenyl ring (C11–C16)] that consolidate the crystal structure (Fig. 6 ▸).

**Figure 7:**
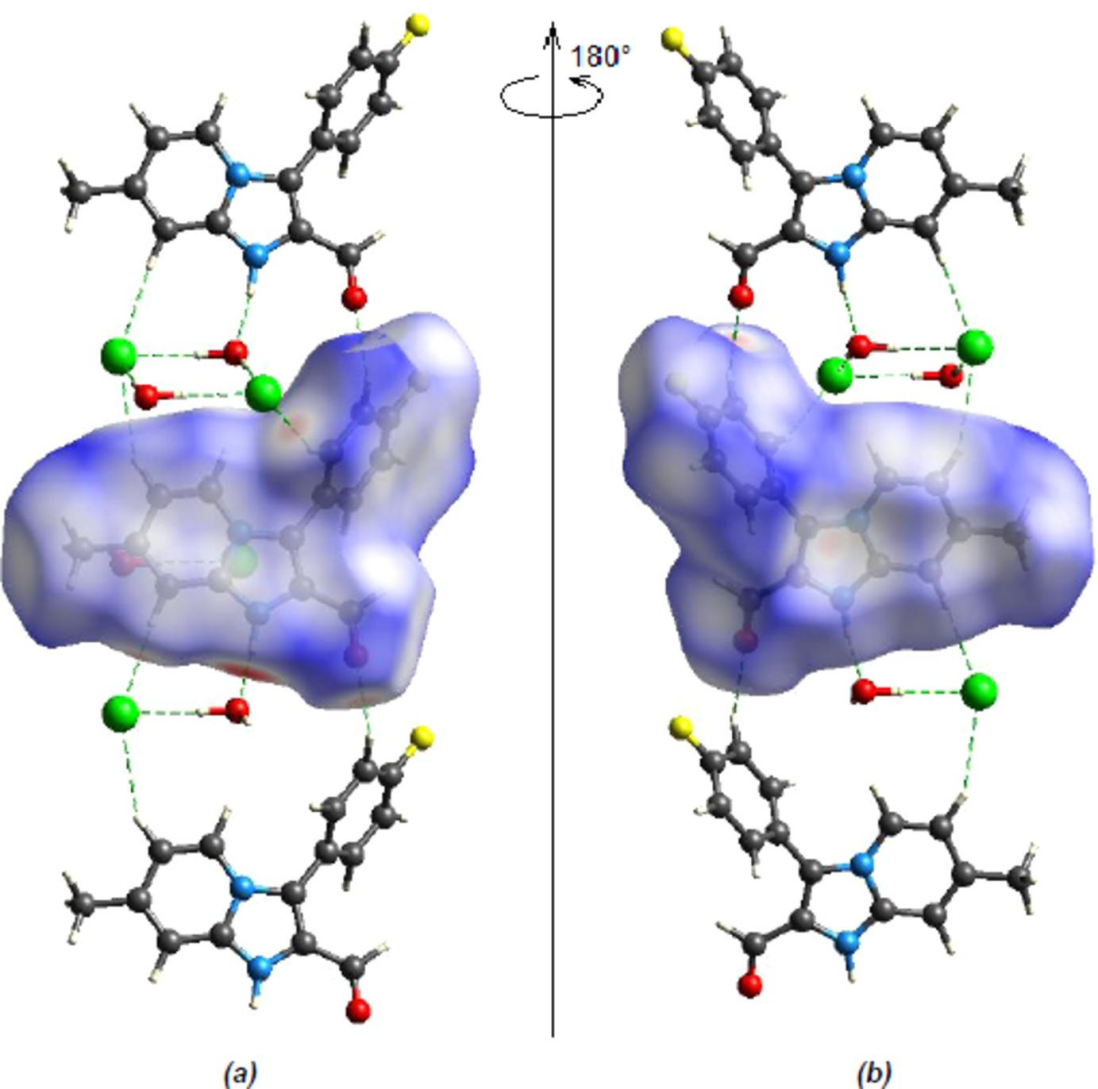
(*a*) Front and (*b*) back sides of the three-dimensional Hirshfeld surface of the title compound mapped over *d*_norm_, with a fixed colour scale from −0.7283 to 1.3376 a.u.

**Figure 8:**
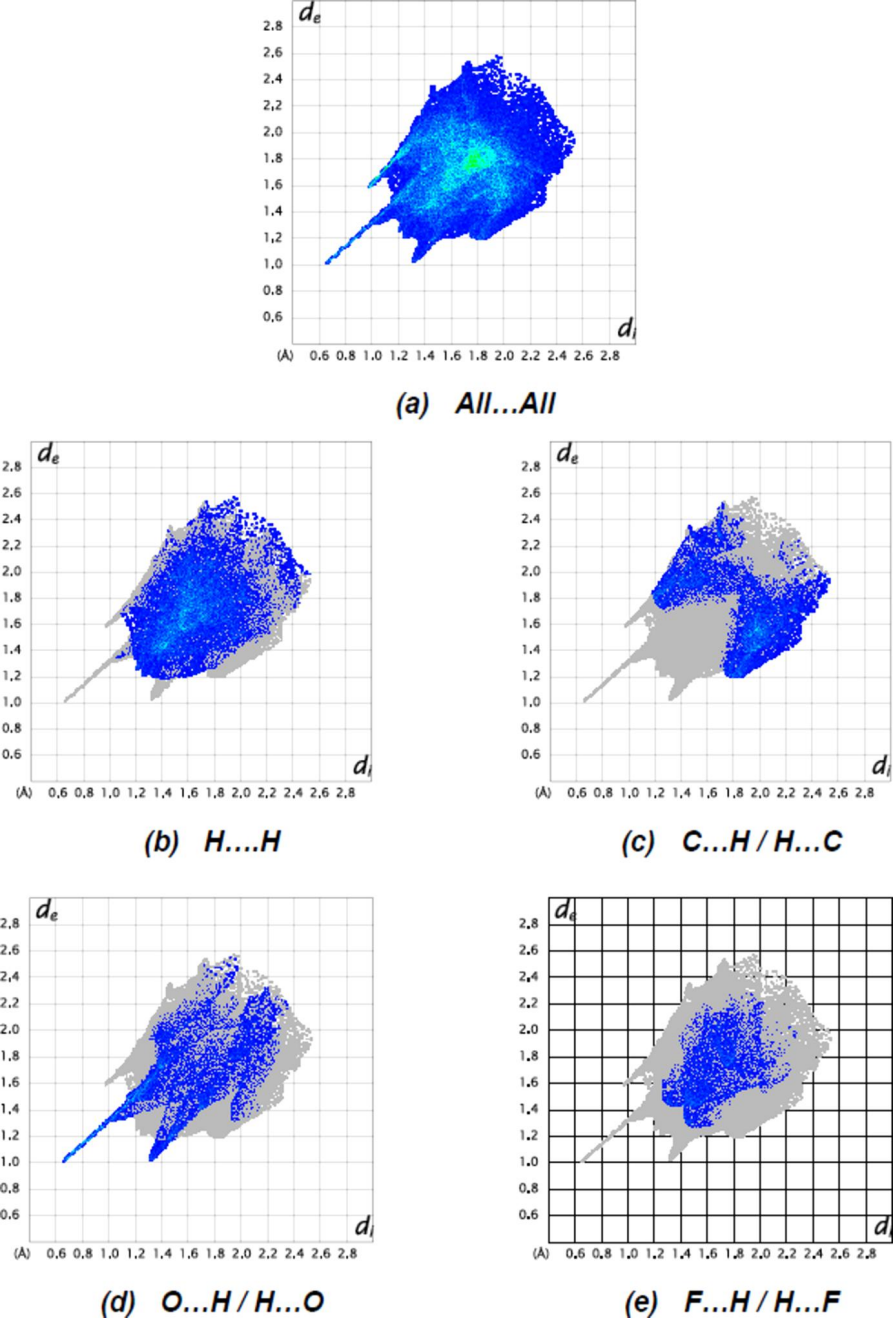
The two-dimensional fingerprint plots of the title compound, showing (*a*) all inter­actions, and delineated into (*b*) H⋯H, (*c*) C⋯H/H⋯C, (*d*) O⋯H/H⋯O and (*e*) F⋯H/H⋯F inter­actions. *d*_e_ and *d*_i_ represent the distances from a point on the Hirshfeld surface to the nearest atoms outside (external) and inside (inter­nal) the surface, respectively.

The Hirshfeld surface mapped over *d*_norm_ was generated using *CrystalExplorer17.5* (Spackman *et al.*, 2021 ▸) with a colour scale from −0.7283 a.u. for red to +1.3376 a.u. for blue. The front and rear views of the Hirshfeld surface mapped over *d*_norm_ are depicted in Fig. 7 ▸. The bright-red circular spots on *d*_norm_ indicate the presence of inter­molecular N1—H1⋯O18^i^, C8—H8⋯Cl1^iv^, C12—H12⋯Cl1^v^ and C13—H13⋯O10^vi^ inter­actions (Table 1 ▸). The percentage contributions from different inter­molecular inter­actions towards the formation of a three-dimensional Hirshfeld surface were computed using two-dimensional fingerprint calculations (Fig. 8 ▸).

Fig. 8 ▸ shows the full two-dimensional fingerprint plots for the mol­ecule and those delineated into the major contacts. H⋯H inter­actions [Fig. 8 ▸(*b*)] are the major contributor (35.2%) to the crystal packing, with C⋯H/H⋯C [Fig. 8 ▸(*c*); 19.0%], O⋯H/H⋯O [Fig. 8 ▸(*d*); 15.5%] and F⋯H/H⋯F [Fig. 8 ▸(*e*); 9.9%] inter­actions representing the next highest contributions. The percentage contributions of comparatively weaker inter­actions are C⋯C (4.6%), N⋯H/H⋯N (2.8%), F⋯O/O⋯F (1.5%), Cl⋯C/C⋯Cl (1.3%), Cl⋯H/H⋯Cl (1.3%), N⋯C/C⋯N (1.3%), F⋯F (1.2%), F⋯C/C⋯F (1.1%) and O⋯O (0.1%). Relevant short inter­molecular atomic contacts are summarized in Table 2 ▸.

The results show that the H⋯H (35.2%) contacts give the major contribution to the crystal packing, and that the C⋯H/H⋯C (19.0%), O⋯H/H⋯O (15.5%) and F⋯H/H⋯F (9.9%) contacts also give a significant contribution to the total area of the Hirshfeld surface.

## 4. Database survey

A search of the Cambridge Structural Database (CSD, Version 5.42, update of September 2021; Groom *et al.*, 2016 ▸) for compounds most closely related to the imidazo[1,2-*a*]pyridin-1-ium unit of the title compound gave the following hits: refcodes LESMAZ (Yin, 2013 ▸), UREPIR (Nichol *et al.*, 2011 ▸), ABAJOE (Rybakov & Babaev, 2011 ▸), BIZWAI02 (Airoldi *et al.*, 2015 ▸), UREYIA (Türkyılmaz *et al.*, 2011 ▸) and NEQPOP (Qiao *et al.*, 2006 ▸).

In the crystal of LESMAZ, the cations and anions are linked into chains parallel to [021] by O—H⋯Cl and N—H⋯Cl hydrogen bonds. In the crystal of UREPIR, N—H⋯O inter­actions form a one-dimensional chain, which propagates in the *b*-axis direction. C—H⋯O inter­actions are also found in the crystal packing. The crystal structure of ABAJOE is consolidated by weak C—H⋯O and C—H⋯Cl inter­actions involving the ‘*olate*’ O atom and the Cl atom attached to the benzoyl group as acceptors. In the crystal of BIZWAI02, mol­ecules are linked by O—H⋯O, N—H⋯O and C—H⋯O hydrogen bonds, and π–π inter­actions [centroid-to-centroid distance = 3.5822 (11) Å], forming a three-dimensional structure. In the crystal of UREYIA, the components are linked by N—H⋯O and C—H⋯O hydrogen bonds and π–π stacking inter­actions [centroid–centroid separation = 3.642 (3) Å]. In the crystal of NEQPOP, inter­molecular O—H⋯O and N—H⋯O hydrogen bonds link the mol­ecules into two-dimensional layers.

## 5. Synthesis and crystallization

A solution of equimolar amounts of 2-amino­pyridine (410 mg, 3.8 mmol) and 2-chloro-2-(di­eth­oxy­meth­yl)-3-(4-fluoro­phen­yl)oxirane (1) or 1-chloro-3,3-dieth­oxy-1-(4-fluoro­phen­yl) propan-2-one (2) (1.05 g, 3.8 mmol) in 25 ml of 95% aqueous ethanol was heated at reflux for 8 h. The solvent was removed *in vacuo*. After purification by column chromatography using a chloro­form/ethyl acetate mixture (3:1 *v*/*v*), 2-(di­eth­oxy­meth­yl)-3-(4-fluoro­phen­yl)imidazo[1,2-*a*]pyridine was ob­tain­ed as a white powder. Gaseous HCl was passed through a solution of 2-(di­eth­oxy­meth­yl)-3-(4-fluoro­phen­yl)imidazo[1,2-*a*]pyridine in chloro­form, leading to the main product, 3-(4-fluoro­phen­yl)-2-formyl-7-methyl­imidazo[1,2-*a*]pyridin-1-ium chloride (**3**) in the form of a white precipitate; this was insoluble in chloro­form and was filtered off and recrystallized from aceto­nitrile (Fig. 1 ▸). Yield 0.61 g (55%); m.p. 509–510 K. Analysis calculated (%) for C_15_H_12_ClFN_2_O: C 70.58, H 4.74, F 7.44, N 10.97, O 6.27; found: C 70.60, H 4.78, F 7.42, N 10.93, O 6.27. ^1^H NMR (300 MHz, DMSO-*d*_6_): δ 2.54 (*s*, 3H, CH_3_), 7.28 (*d*, *J* = 6.6 Hz, 1H, 6CH), 7.55 (*dd*, *J* = 8.8, 5.5 Hz, 2H, Ar), 7.75 (*s*, 1H, NH), 7.90 (*dd*, *J* = 8.6, 5.5 Hz, 2H, Ar), 8.35 (*s*, 1H, 8CH), 8.47 (*d*, *J* = 7.1 Hz, 1H, 5CH), 9.85 (*s*, 1H, CHO). ^13^C NMR (200 MHz, DMSO-*d*_6_): δ 21.27, 111.92, 116.44, 116.87, 119.67, 120.02, 126.18, 130.39, 131.39, 133.59 (*d*, *J* = 35 Hz, CF), 141.40, 147.07, 161.11, 166.06, 182.45. ESI–MS: *m*/*z*: 255.0928 [*M* + H]^+^.

## 6. Refinement

Crystal data, data collection and structure refinement details are summarized in Table 3 ▸. The N-bound H atom and the H atoms of the water mol­ecule were located in a difference Fourier map and refined freely along with their isotropic displacement parameters. C-bound H atoms were included in calculated positions and treated as riding atoms (C—H = 0.95–0.98 Å), with *U*_iso_(H) = 1.2*U*_eq_(C) for aromatic H atoms and 1.5*U*_eq_(C) for methyl H atoms.

## Supplementary Material

Crystal structure: contains datablock(s) I. DOI: 10.1107/S2056989023007272/jy2034sup1.cif

Structure factors: contains datablock(s) I. DOI: 10.1107/S2056989023007272/jy2034Isup2.hkl

Supporting information file. DOI: 10.1107/S2056989023007272/jy2034Isup3.cml

CCDC reference: 2289534

Additional supporting information:  crystallographic information; 3D view; checkCIF report

## Figures and Tables

**Figure 4:**
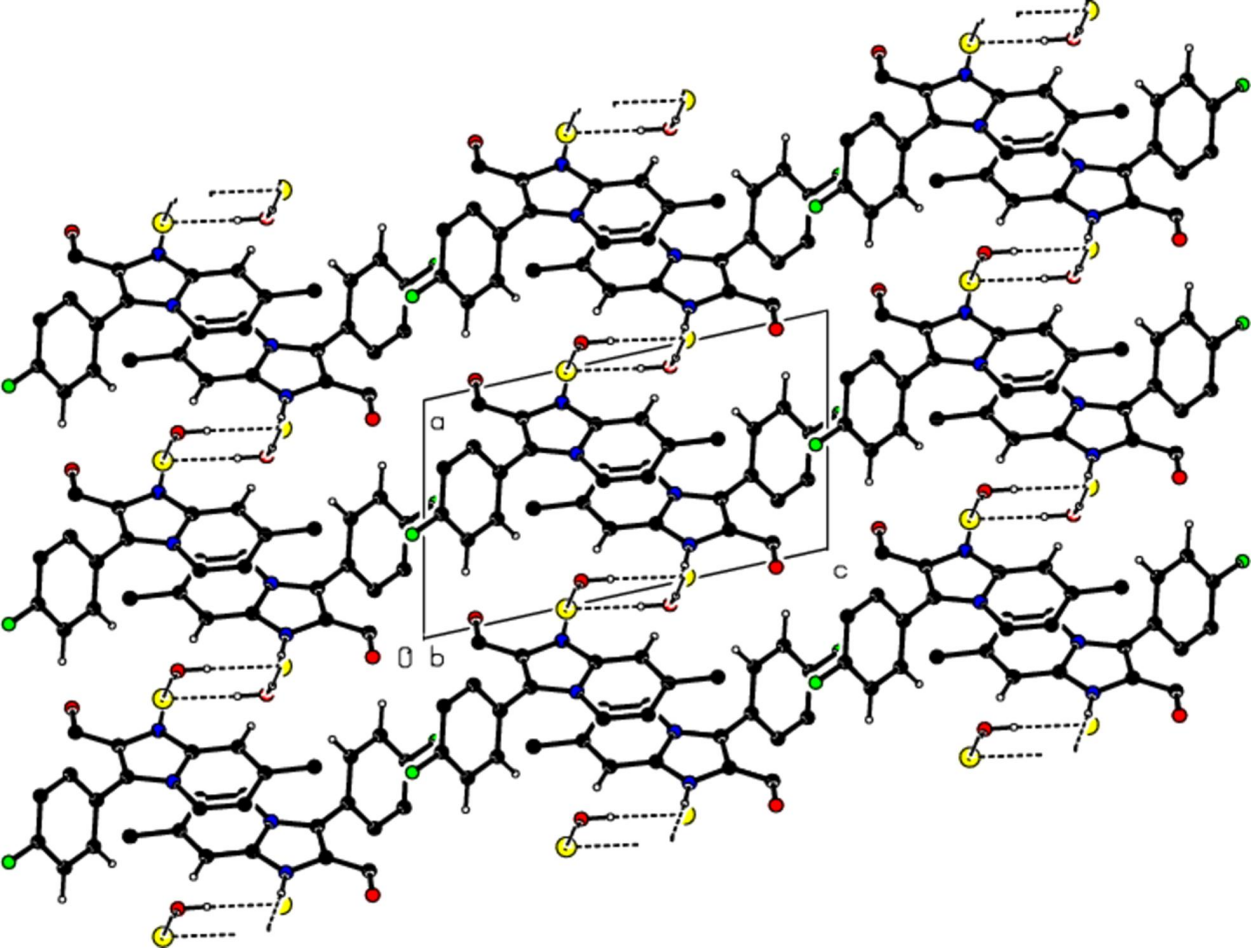
View of the mol­ecular packing along the *b* axis. Hydrogen bonds are depicted as in Fig. 3 ▸.

**Table 1:** Hydrogen-bond geometry (Å, °)

*D*—H⋯*A*	*D*—H	H⋯*A*	*D*⋯*A*	*D*—H⋯*A*
N1—H1⋯O18^i^	0.91 (2)	1.77 (2)	2.6754 (16)	174 (2)
O18—H18*A*⋯Cl1^ii^	0.87 (2)	2.24 (2)	3.1070 (11)	175 (2)
O18—H18*B*⋯Cl1^iii^	0.87 (2)	2.24 (2)	3.1142 (11)	178.0 (19)
C8—H8⋯Cl1^iv^	0.95	2.69	3.6431 (15)	176
C12—H12⋯Cl1^v^	0.95	2.71	3.5610 (16)	150
C13—H13⋯O10^vi^	0.95	2.41	3.057 (2)	125

Symmetry codes: (i) 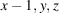 ; (ii) 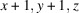 ; (iii) 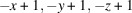 ; (iv) 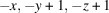 ; (v) 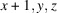 ; (vi) 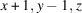 .

**Table 2:** Summary of short inter­atomic contacts (Å) in the title compound

Contact	Distance	Symmetry operation
H13⋯O10	2.41	*x* + 1, *y* − 1, *z*
F1⋯H17*C*	2.78	*x*, *y* − 1, *z* + 1
H9⋯F1	2.79	−*x* + 1, −*y*, −*z* + 2
H9⋯O10	2.71	−*x*, −*y* + 1, −*z* + 2
H16⋯Cl1	3.04	*x*, *y*, *z*
H17*B*⋯C2	3.02	−*x* + 1, −*y* + 1, −*z* + 1
H5⋯C6	3.02	−*x* + 1, −*y*, −*z* + 1
H17*A*⋯O18	2.78	−*x* + 1, −*y* + 1, −*z* + 1
H8⋯Cl1	2.69	−*x*, −*y* + 1, −*z* + 1
H15⋯C9	3.08	−*x*, −*y*, −*z* + 2
H12⋯Cl1	2.71	*x* + 1, *y*, *z*
H5⋯O18	2.76	*x*, *y* − 1, *z*
Cl1⋯H6	2.94	−*x* + 1, −*y*, −*z* + 1
H18*A*⋯Cl1	2.24	*x* + 1, *y* + 1, *z*
O18⋯H1	1.77	*x* + 1, *y*, *z*

**Table 3:** Experimental details

Crystal data	
Chemical formula	C_15_H_12_FN_2_O^+^·Cl^−^·H_2_O
*M*_r_	308.73
Crystal system, space group	Triclinic, *P* 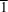
Temperature (K)	100
*a*, *b*, *c* (Å)	7.45681 (13), 8.41737 (10), 12.8928 (2)
α, β, γ (°)	74.0382 (12), 73.7634 (14), 72.7034 (13)
*V* (Å^3^)	725.40 (2)
*Z*	2
Radiation type	Cu *K*α
μ (mm^−1^)	2.50
Crystal size (mm)	0.33 × 0.19 × 0.15

Data collection	
Diffractometer	Rigaku XtaLAB Synergy Dualflex diffractometer with a HyPix detector
Absorption correction	Gaussian (*CrysAlis PRO*; Rigaku OD, 2023 ▸)
*T*_min_, *T*_max_	0.404, 1.000
No. of measured, independent and observed [*I* > 2σ(*I*)] reflections	15845, 3082, 3033
*R*_int_	0.027
(sin θ/λ)_max_ (Å^−1^)	0.634

Refinement	
*R*[*F* ^2^ > 2σ(*F* ^2^)], *wR*(*F* ^2^), *S*	0.033, 0.086, 1.03
No. of reflections	3082
No. of parameters	203
H-atom treatment	H atoms treated by a mixture of independent and constrained refinement
Δρ_max_, Δρ_min_ (e Å^−3^)	0.34, −0.24

Computer programs: *CrysAlis PRO* (Rigaku OD, 2023 ▸), *SHELXT2019* (Sheldrick, 2015*a* ▸), *SHELXL2019* (Sheldrick, 2015*b* ▸), *ORTEP-3 for Windows* (Farrugia, 2012 ▸) and *PLATON* (Spek, 2020 ▸).

## supplementary crystallographic information

### Crystal data

**Table:**

C_15_H_12_FN_2_O^+^·Cl^−^·H_2_O	*Z* = 2
*M**_r_* = 308.73	*F*(000) = 320
Triclinic, *P*1	*D*_x_ = 1.413 Mg m^−^^3^
*a* = 7.45681 (13) Å	Cu *K*α radiation, λ = 1.54184 Å
*b* = 8.41737 (10) Å	Cell parameters from 11896 reflections
*c* = 12.8928 (2) Å	θ = 3.6–77.3°
α = 74.0382 (12)°	µ = 2.50 mm^−^^1^
β = 73.7634 (14)°	*T* = 100 K
γ = 72.7034 (13)°	Prism, colorless
*V* = 725.40 (2) Å^3^	0.33 × 0.19 × 0.15 mm

### Data collection

**Table:**

Rigaku XtaLAB Synergy Dualflex diffractometer with a HyPix detector	3082 independent reflections
Radiation source: micro-focus sealed X-ray tube, PhotonJet (Cu) X-ray Source	3033 reflections with *I* > 2σ(*I*)
Mirror monochromator	*R*_int_ = 0.027
Detector resolution: 10.0000 pixels mm^-1^	θ_max_ = 77.9°, θ_min_ = 3.7°
ω scans	*h* = −9→9
Absorption correction: gaussian (CrysAlis PRO; Rigaku OD, 2023)	*k* = −10→9
*T*_min_ = 0.404, *T*_max_ = 1.000	*l* = −16→16
15845 measured reflections	

### Refinement

**Table:**

Refinement on *F*^2^	Primary atom site location: dual
Least-squares matrix: full	Secondary atom site location: difference Fourier map
*R*[*F*^2^ > 2σ(*F*^2^)] = 0.033	Hydrogen site location: mixed
*wR*(*F*^2^) = 0.086	H atoms treated by a mixture of independent and constrained refinement
*S* = 1.03	*w* = 1/[σ^2^(*F*_o_^2^) + (0.0415*P*)^2^ + 0.4038*P*] where *P* = (*F*_o_^2^ + 2*F*_c_^2^)/3
3082 reflections	(Δ/σ)_max_ = 0.001
203 parameters	Δρ_max_ = 0.34 e Å^−^^3^
0 restraints	Δρ_min_ = −0.24 e Å^−^^3^

### Special details

**Table:**

Geometry. All esds (except the esd in the dihedral angle between two l.s. planes) are estimated using the full covariance matrix. The cell esds are taken into account individually in the estimation of esds in distances, angles and torsion angles; correlations between esds in cell parameters are only used when they are defined by crystal symmetry. An approximate (isotropic) treatment of cell esds is used for estimating esds involving l.s. planes.

### Fractional atomic coordinates and isotropic or equivalent isotropic displacement parameters (Å^2^)

**Table:**

	*x*	*y*	*z*	*U*_iso_*/*U*_eq_	
Cl1	0.01542 (4)	0.11097 (4)	0.64700 (3)	0.02403 (11)	
F1	0.56045 (14)	−0.35098 (11)	1.02878 (8)	0.0367 (2)	
O18	0.90723 (15)	0.80467 (12)	0.61002 (9)	0.0241 (2)	
N4	0.37156 (15)	0.27422 (14)	0.62115 (9)	0.0190 (2)	
N1	0.14182 (17)	0.49767 (15)	0.65794 (10)	0.0215 (2)	
C8A	0.26290 (18)	0.42655 (16)	0.57537 (11)	0.0196 (3)	
C11	0.37885 (19)	0.08741 (17)	0.81151 (11)	0.0210 (3)	
C5	0.51012 (19)	0.17210 (16)	0.55569 (12)	0.0214 (3)	
H5	0.586532	0.067821	0.587952	0.026*	
C12	0.5714 (2)	0.03302 (17)	0.81846 (11)	0.0233 (3)	
H12	0.660070	0.098082	0.773040	0.028*	
C3	0.30886 (19)	0.24618 (17)	0.73582 (11)	0.0208 (3)	
C7	0.4192 (2)	0.37812 (17)	0.39448 (11)	0.0228 (3)	
C2	0.16899 (19)	0.38788 (17)	0.75689 (11)	0.0217 (3)	
C8	0.28524 (19)	0.48118 (17)	0.46032 (11)	0.0215 (3)	
H8	0.209704	0.586666	0.428855	0.026*	
C14	0.5015 (2)	−0.20644 (18)	0.95627 (12)	0.0267 (3)	
C16	0.2482 (2)	−0.00887 (19)	0.87709 (12)	0.0271 (3)	
H16	0.117247	0.027457	0.871462	0.032*	
C6	0.5351 (2)	0.22342 (17)	0.44449 (12)	0.0232 (3)	
H6	0.631903	0.154967	0.398747	0.028*	
C13	0.6343 (2)	−0.11584 (18)	0.89146 (12)	0.0256 (3)	
H13	0.765513	−0.154250	0.896619	0.031*	
C17	0.4406 (2)	0.4222 (2)	0.27152 (12)	0.0295 (3)	
H17A	0.374708	0.355153	0.250243	0.044*	
H17B	0.577414	0.396555	0.235805	0.044*	
H17C	0.383705	0.543651	0.248116	0.044*	
C15	0.3103 (2)	−0.1578 (2)	0.95051 (12)	0.0303 (3)	
H15	0.223073	−0.224471	0.995677	0.036*	
C9	0.0719 (2)	0.4320 (2)	0.86403 (12)	0.0305 (3)	
H9	0.092957	0.349329	0.929217	0.037*	
H18A	0.937 (3)	0.887 (3)	0.6243 (19)	0.046 (6)*	
H18B	0.926 (3)	0.830 (3)	0.538 (2)	0.045 (6)*	
H1	0.059 (3)	0.602 (3)	0.6466 (17)	0.042 (5)*	
O10	−0.0336 (2)	0.56891 (18)	0.87279 (11)	0.0572 (4)	

### Atomic displacement parameters (Å^2^)

**Table:**

	*U*^11^	*U*^22^	*U*^33^	*U*^12^	*U*^13^	*U*^23^
Cl1	0.02505 (17)	0.02388 (17)	0.02491 (17)	−0.00803 (12)	−0.00593 (12)	−0.00495 (12)
F1	0.0455 (6)	0.0280 (5)	0.0283 (5)	−0.0034 (4)	−0.0120 (4)	0.0055 (4)
O18	0.0284 (5)	0.0197 (5)	0.0241 (5)	−0.0053 (4)	−0.0058 (4)	−0.0045 (4)
N4	0.0200 (5)	0.0177 (5)	0.0196 (5)	−0.0050 (4)	−0.0035 (4)	−0.0047 (4)
N1	0.0221 (5)	0.0188 (5)	0.0217 (6)	−0.0011 (4)	−0.0052 (4)	−0.0050 (4)
C8A	0.0195 (6)	0.0174 (6)	0.0228 (6)	−0.0051 (5)	−0.0046 (5)	−0.0048 (5)
C11	0.0231 (6)	0.0203 (6)	0.0189 (6)	−0.0031 (5)	−0.0039 (5)	−0.0057 (5)
C5	0.0201 (6)	0.0178 (6)	0.0265 (7)	−0.0047 (5)	−0.0032 (5)	−0.0068 (5)
C12	0.0236 (6)	0.0232 (6)	0.0216 (6)	−0.0041 (5)	−0.0030 (5)	−0.0060 (5)
C3	0.0214 (6)	0.0215 (6)	0.0205 (6)	−0.0060 (5)	−0.0045 (5)	−0.0048 (5)
C7	0.0267 (7)	0.0225 (6)	0.0220 (6)	−0.0126 (5)	−0.0023 (5)	−0.0048 (5)
C2	0.0218 (6)	0.0223 (6)	0.0202 (6)	−0.0044 (5)	−0.0047 (5)	−0.0036 (5)
C8	0.0245 (6)	0.0187 (6)	0.0227 (6)	−0.0070 (5)	−0.0064 (5)	−0.0031 (5)
C14	0.0356 (8)	0.0216 (7)	0.0194 (6)	−0.0015 (6)	−0.0077 (6)	−0.0026 (5)
C16	0.0249 (7)	0.0286 (7)	0.0261 (7)	−0.0068 (6)	−0.0051 (5)	−0.0032 (6)
C6	0.0242 (6)	0.0218 (6)	0.0247 (7)	−0.0073 (5)	−0.0006 (5)	−0.0094 (5)
C13	0.0260 (7)	0.0250 (7)	0.0235 (7)	0.0007 (5)	−0.0069 (5)	−0.0075 (5)
C17	0.0375 (8)	0.0301 (7)	0.0219 (7)	−0.0135 (6)	−0.0021 (6)	−0.0054 (6)
C15	0.0330 (8)	0.0305 (8)	0.0244 (7)	−0.0113 (6)	−0.0036 (6)	0.0006 (6)
C9	0.0306 (7)	0.0321 (8)	0.0227 (7)	0.0010 (6)	−0.0039 (6)	−0.0073 (6)
O10	0.0698 (10)	0.0467 (8)	0.0304 (6)	0.0263 (7)	−0.0082 (6)	−0.0152 (6)

### Geometric parameters (Å, º)

**Table:**

F1—C14	1.3542 (16)	C7—C8	1.3734 (19)
O18—H18A	0.86 (2)	C7—C6	1.428 (2)
O18—H18B	0.87 (2)	C7—C17	1.4988 (19)
N4—C8A	1.3719 (17)	C2—C9	1.4605 (19)
N4—C5	1.3791 (17)	C8—H8	0.9500
N4—C3	1.3955 (17)	C14—C13	1.377 (2)
N1—C8A	1.3411 (17)	C14—C15	1.379 (2)
N1—C2	1.3811 (17)	C16—H16	0.9500
N1—H1	0.92 (2)	C16—C15	1.389 (2)
C8A—C8	1.4042 (19)	C6—H6	0.9500
C11—C12	1.3920 (19)	C13—H13	0.9500
C11—C3	1.4709 (18)	C17—H17A	0.9800
C11—C16	1.3973 (19)	C17—H17B	0.9800
C5—H5	0.9500	C17—H17C	0.9800
C5—C6	1.354 (2)	C15—H15	0.9500
C12—H12	0.9500	C9—H9	0.9500
C12—C13	1.387 (2)	C9—O10	1.2011 (19)
C3—C2	1.3654 (19)		
			
H18A—O18—H18B	103 (2)	C8A—C8—H8	120.9
C8A—N4—C5	121.11 (12)	C7—C8—C8A	118.26 (12)
C8A—N4—C3	108.76 (11)	C7—C8—H8	120.9
C5—N4—C3	130.04 (11)	F1—C14—C13	118.85 (13)
C8A—N1—C2	108.37 (11)	F1—C14—C15	118.03 (13)
C8A—N1—H1	123.1 (13)	C13—C14—C15	123.12 (13)
C2—N1—H1	128.5 (13)	C11—C16—H16	120.1
N4—C8A—C8	121.05 (12)	C15—C16—C11	119.86 (13)
N1—C8A—N4	108.00 (11)	C15—C16—H16	120.1
N1—C8A—C8	130.92 (12)	C5—C6—C7	121.48 (12)
C12—C11—C3	121.05 (12)	C5—C6—H6	119.3
C12—C11—C16	120.14 (13)	C7—C6—H6	119.3
C16—C11—C3	118.81 (12)	C12—C13—H13	120.9
N4—C5—H5	120.7	C14—C13—C12	118.22 (13)
C6—C5—N4	118.69 (12)	C14—C13—H13	120.9
C6—C5—H5	120.7	C7—C17—H17A	109.5
C11—C12—H12	119.9	C7—C17—H17B	109.5
C13—C12—C11	120.26 (13)	C7—C17—H17C	109.5
C13—C12—H12	119.9	H17A—C17—H17B	109.5
N4—C3—C11	123.90 (11)	H17A—C17—H17C	109.5
C2—C3—N4	105.71 (11)	H17B—C17—H17C	109.5
C2—C3—C11	130.28 (12)	C14—C15—C16	118.39 (14)
C8—C7—C6	119.34 (13)	C14—C15—H15	120.8
C8—C7—C17	121.11 (13)	C16—C15—H15	120.8
C6—C7—C17	119.51 (13)	C2—C9—H9	118.8
N1—C2—C9	122.80 (12)	O10—C9—C2	122.45 (14)
C3—C2—N1	109.06 (12)	O10—C9—H9	118.8
C3—C2—C9	127.88 (13)		
			
F1—C14—C13—C12	−179.27 (12)	C12—C11—C3—N4	62.54 (18)
F1—C14—C15—C16	179.42 (13)	C12—C11—C3—C2	−121.88 (16)
N4—C8A—C8—C7	−0.07 (19)	C12—C11—C16—C15	1.0 (2)
N4—C5—C6—C7	−1.2 (2)	C3—N4—C8A—N1	3.08 (14)
N4—C3—C2—N1	1.89 (15)	C3—N4—C8A—C8	−175.22 (12)
N4—C3—C2—C9	−172.39 (14)	C3—N4—C5—C6	175.22 (12)
N1—C8A—C8—C7	−177.93 (13)	C3—C11—C12—C13	179.08 (12)
N1—C2—C9—O10	−2.3 (3)	C3—C11—C16—C15	−178.93 (13)
C8A—N4—C5—C6	−1.22 (18)	C3—C2—C9—O10	171.26 (17)
C8A—N4—C3—C11	173.45 (12)	C2—N1—C8A—N4	−1.87 (15)
C8A—N4—C3—C2	−3.04 (14)	C2—N1—C8A—C8	176.21 (13)
C8A—N1—C2—C3	−0.05 (15)	C8—C7—C6—C5	3.0 (2)
C8A—N1—C2—C9	174.58 (13)	C16—C11—C12—C13	−0.8 (2)
C11—C12—C13—C14	−0.2 (2)	C16—C11—C3—N4	−117.56 (15)
C11—C3—C2—N1	−174.29 (13)	C16—C11—C3—C2	58.0 (2)
C11—C3—C2—C9	11.4 (2)	C6—C7—C8—C8A	−2.29 (19)
C11—C16—C15—C14	−0.1 (2)	C13—C14—C15—C16	−1.0 (2)
C5—N4—C8A—N1	−179.80 (11)	C17—C7—C8—C8A	175.47 (12)
C5—N4—C8A—C8	1.90 (18)	C17—C7—C6—C5	−174.78 (13)
C5—N4—C3—C11	−3.3 (2)	C15—C14—C13—C12	1.2 (2)
C5—N4—C3—C2	−179.83 (13)		

### Hydrogen-bond geometry (Å, º)

**Table:**

*D*—H···*A*	*D*—H	H···*A*	*D*···*A*	*D*—H···*A*
N1—H1···O18^i^	0.91 (2)	1.77 (2)	2.6754 (16)	174 (2)
O18—H18*A*···Cl1^ii^	0.87 (2)	2.24 (2)	3.1070 (11)	175 (2)
O18—H18*B*···Cl1^iii^	0.87 (2)	2.24 (2)	3.1142 (11)	178.0 (19)
C8—H8···Cl1^iv^	0.95	2.69	3.6431 (15)	176
C12—H12···Cl1^v^	0.95	2.71	3.5610 (16)	150
C13—H13···O10^vi^	0.95	2.41	3.057 (2)	125

Symmetry codes: (i) *x*−1, *y*, *z*; (ii) *x*+1, *y*+1, *z*; (iii) −*x*+1, −*y*+1, −*z*+1; (iv) −*x*, −*y*+1, −*z*+1; (v) *x*+1, *y*, *z*; (vi) *x*+1, *y*−1, *z*.
